# Protein phosphatase 2A interacts with Verthandi/Rad21 to regulate mitosis and organ development in *Drosophila*

**DOI:** 10.1038/s41598-019-44027-3

**Published:** 2019-05-20

**Authors:** Lee-Hyang Kim, Sung-Tae Hong, Kwang-Wook Choi

**Affiliations:** 1 Department of Biological Sciences, Korea Advanced Institute of Science and Technology (KAIST), Daejeon, Republic of Korea; 2 Department of Anatomy & Cell Biology, College of Medicine, Chungnam National University, Daejeon, Republic of Korea; 3 Department of Medical Science, College of Medicine, Chungnam National University, Daejeon, Republic of Korea

**Keywords:** Developmental biology, Organogenesis

## Abstract

Rad21/Scc1 is a subunit of the cohesin complex implicated in gene regulation as well as sister chromatid cohesion. The level of Rad21/Scc1 must be controlled for proper mitosis and gene expression during development. Here, we identify the PP2A catalytic subunit encoded by *microtubule star* (*mts*) as a regulator of *Drosophila* Rad21/Verthandi (Vtd). Mutations in *mts* and *vtd* cause synergistic mitotic defects, including abnormal spindles and loss of nuclei during nuclear division in early embryo. Depletion of Mts and Vtd in developing wing synergistically reduces the Cut protein level, causing severe defects in wing growth. Mts and PP2A subunit Twins (Tws) interact with Vtd protein. Loss of Mts or Tws reduces Vtd protein level. Reduced proteasome function suppresses mitotic defects caused by mutations in *mts* and *vtd*. Taken together, this work provides evidence that PP2A is required for mitosis and wing growth by regulating the Vtd level through the proteasomal pathway.

## Introduction

Phosphorylation is a key posttranslational mechanism for the regulation of cell signaling pathways. Increasing evidence indicates that dephosphorylation also plays pivotal roles to control diverse developmental processes, including cell growth, proliferation and cell polarity^1–5^. Protein phosphatase 2A (PP2A) is one of the major evolutionarily conserved serine/threonine phosphatases in eukaryotes^6,7^ that functions as a heterotrimeric complex composed of three subunits: catalytic (C), structural (A) and regulatory (B) subunits^1^. In *Drosophila*, the C subunit of PP2A was identified as an essential factor for mitosis. The C subunit was named Microtubule star (Mts) because *mts* mutant embryos show abnormal pattern of microtubule spindles emanating in all directions from centrosomes during mitosis^8^.

With respect to the function of Mts in mitosis, it is noteworthy that a cohesin subunit Verthadi (Vtd) was isolated as an interacting partner of Mts from a two-hybrid screen described in this study. Vtd is an ortholog of the Rad21/Scc1 subunit in the cohesin complex that controls separation and cohesion of sister chromatids during mitosis^9–11^. Cohesin is an evolutionarily conserved complex consisting of four subunits, Rad21/Scc1, Stromalin (SA) and Structural Maintenance of Chromosome 1 and 3 (SMC1 and SMC3)^12–14^. Cohesin forms a ring-like structure that holds sister chromatids after DNA replication^15,16^. Rad21/Scc1 is phosphorylated by Polo kinase and cleaved by separase, leading to the release of sister chromatids during anaphase in yeast^9,17^. However, Polo-dependent regulation of Rad21 has not yet been confirmed in *Drosophila*.

Although the core components of a cohesin ring are conserved in eukaryotes, mechanisms underlying cohesin regulation are complicated with many variations^18^. In yeasts, cohesin is released in one step along the chromosome lengths during the metaphase/anaphase transition. In metazoan mitosis, however, cohesin is released in two steps. Firstly, cohesin is released from the arms of sister chromatids during prophase and prometaphase by phosphorylation of the SA2 cohesin subunit. During this step, centromeric cohesin is protected from cleavage by Shugosin (Sgo) and PP2A. In the second step, centromeric cohesin is released by cleavage of the Rad21/Scc1 subunit during the metaphase/anaphase transition^19,20^. In addition to the traditional view of the cohesin function on sister chromatids, cohesin also functions at spindle poles and centrosomes^11,21–24^.

In *Drosophila* S2 cells, depletion of Vtd results in abnormal chromosome and spindle morphology with premature sister chromatid separation^10^, consistent with the role of Rad21/Scc1 for stabilizing the cohesin complex. Vtd is not required for sister chromatid cohesion in meiosis^25,26^, suggesting that Vtd is specifically involved in mitotic segregation of sister chromatids. A systematic RNAi screen in cultured S2 *Drosophila* cells has found the roles of multiple protein phosphatases including PP2A in mitosis^27^. S2 cells depleted in Mts or other PP2A subunits show aberrant arrays of microtubules as seen in *mts* mutant embryos. Widerborst (Wdb) (*Drosophila* B56 subunit) forms a protein complex with MEI-S332 (*Drosophila* homolog of Sgo) at the centromeres as in human and yeast cells. Whereas Sgo recruits PP2A in yeasts, Wdb is required for the localization of MEI-S332 but not *vice versa* as in mammalian cells. Although Sgo is crucial for protecting cohesion during meiosis in all eukaryotes tested, MEI-S322 is not essential for mitosis in *Drosophila*^28^. Based on these differences between organisms, it is important to identify specific function of PP2A and Vtd/Rad21 in *Drosophila* to understand their functional relationship^19,29–33^. A recent study has shown that the Dalmatian (Dmt), a *Drosophila* ortholog of the vertebrate cohesin-interacting Sororin, is required for recruiting PP2A to the pericentric heterochromatin in S2 cell. Interestingly, Dmt can restore mitotic defects of Sgo1-depleted human cells, indicating that Dmt has cohesin protection activity^34^. However, it is unknown whether Dmt and/or PP2A are required for the cohesin function in developing fly tissues and whether these proteins are involved in regulating the stability and the level of Cohesin complex proteins.

It has been suggested that a major role of Mts in early *Drosophila* embryo is to link spindle microtubules to kinetochore since loss of Mts leads to spindle microtubules formed in random directions from centrosomes without association with chromosomes. Another intriguing phenotype of *mts* loss-of-function mutations involves frequent loss of mitotic nuclei, resulting in free centrosomes separated from nuclei^8^. Similar phenotypes were also found in early embryos of *abnormal anaphase resolution* (*aar*) mutants, which are allelic to *twins* (*tws*), the B subunit of PP2A^35^. It is unknown whether cohesin subunit mutants also show similar defects in *Drosophila embryos*.

In addition to the function in mitosis, cohesin has additional roles in transcriptional gene regulation. Nipped-B is a *Drosophila* adherin that is required for loading cohesin onto chromosomes. Mutations in *Nipped-B* affect gene expression resulting in developmental and neurological defects that might model the Cornelia de Lange Syndrome caused by mutations in NIPBL, the human homolog of Nipped-B^36^. Nipped-B and cohesin are involved in regulating the expression of the *cut* gene that is essential for wing outgrowth^37,38^. Along with these findings, it has been shown that Vtd belongs to the trithorax group proteins that facilitate transcription for gene regulation^39,40^. Mitosis and organ growth must be regulated for normal development. Despite the importance of Vtd in these events, it is unknown how the level of Vtd protein is regulated in developing animals.

In this study, we analyze molecular and functional relationships between Mts and Vtd in *Drosophila*. Mutations or knockdown of *mts* and *vtd* cause synergistic spindle defects and nuclear loss during nuclear division in early embryo. *mts* and *vtd* also show strong genetic interaction in developing wing. We demonstrate physical interaction between Mts and Vtd. Further, we provide evidence that PP2A is required for the maintenance of Vtd protein level by a proteasome-dependent pathway. Hence, we propose that Mts is required for Vtd stability to control mitosis and wing growth.

## Results

### Vtd is a direct interacting partner for Mts

**Figure 1:**
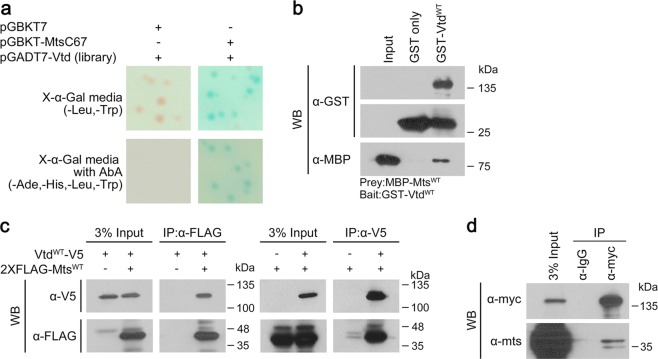
Mts physically interacts with Vtd. (**a**) Confirmation of positive interaction between C-terminal part of Mts (MtsC67) and Vtd fragment by yeast two-hybrid assay. Vtd alone shows no lacZ-positive blue colonies on the media plate lacking leucine and tryptophan (-Leu/-Trp/X-α-Gal plate) or the media deficient in adenine, histidine, leucine and tryptophan (-Ade/-His/-Leu/-Trp/X-α-Gal/Aureobasidin A(AbA)). Direct interaction between MtsC67 and Vtd results in the formation of blue colonies on both selection media plates. (**b)** GST pull-down assay with MBP-Mts^WT^ and GST-Vtd^WT^. Mts and Vtd show direct interaction *in vitro*. (**c**) Co-immunoprecipitation assay using S2 cell extract. FLAG-tagged Mts^WT^ forms a protein complex with V5-tagged Vtd^WT^ in S2 cell. (**d**) Co-immunoprecipitation assay using embryo extract. Embryos are *vtd* mutants rescued by wild-type Vtd-Myc (w*; *vtd*^*ex3*^, *tub::vtd*^*WT*^*-myc*_10_). Myc-tagged Vtd^WT^ co-immunoprecipitates with endogenous Mts^WT^ in embryos. (**b**–**d**) Full-length blots are presented in Supplementary Fig. 6.

To isolate protein partners that interact with Mts, we screened a yeast two-hybrid library for *Drosophila* using a C-terminal Mts region (amino acid 243~309, MtsC67) that showed a low level of autoactivation. From this screen, we obtained three interacting partners for Mts; Verthandi (Vtd) (Fig. 1a), Replication Protein A 70 (RPA70), and CG3262. Among these proteins, we further characterized the interaction between Mts and Vtd because both proteins play roles in mitosis^8,10,11,17,27,39,41^. Vtd/Rad21 was also in a PP2A-interacting network from a *Drosophila* proteomic analysis by mass-spectrometry, although it was not determined whether Vtd directly binds to PP2A^42^.

First, we confirmed the physical interaction between Mts and Vtd. *In vitro* GST (Glutathione-S-transferase) pull down assay using MBP (Maltose binding protein)-tagged full-length Mts and GST-tagged full-length Vtd showed that Mts directly binds to Vtd protein (Fig. 1b). This interaction was verified by co-immunoprecipitation (co-IP) using S2 cells transfected with 2xFLAG-tagged-Mts and V5-tagged-Vtd (Fig. 1c). In reciprocal co-IP experiments using FLAG or V5 antibody, Mts protein formed a protein complex with Vtd in S2 cells. Next, we tested whether Mts and Vtd interact in physiological conditions *in vivo*. We performed co-immunoprecipitaion (co-IP) using extracts from *vtd* mutant embryos expressing transgenic *vtd* wild-type gene (Genotype: w*; vtd^*ex3*^*, tub::vtd*^*WT*^*-myc*_*10*_). Vtd^WT^-myc_10_ expressed by tubulin promoter rescues *vtd*^*ex3*^ mutant phenotypes^43^. This co-IP assay showed that Myc-tagged-Vtd proteins form a protein complex *in vivo* with endogenous Mts (Fig. 1d).

Since three separate regions of Vtd are conserved in eukayotic Rad21 family proteins, we tested whether Mts interacts with these conserved domains of Vtd. We used the Eukaryotic Linear Motif (ELM) resource (http://elm.eu.org/) to identify distinct structural domains of Vtd. According to the ELM analysis, Vtd can be divided into five domains (Supplementary Fig. S1a). Vtd^1~152^, Vtd^273~530^ and Vtd^625~715^ are globluar domains that show strong homology to Rad21/Scc1 (Supplementary Fig. S1a,b). The remaining two regions (153–272 and 531–624) are not conserved and their functions are unknown. We performed co-IP assays with 2xFLAG-tagged full-length Mts and truncated Vtd fragments tagged with V5 expressed in S2 cells. Co-IP results indicated that three Vtd regions (Vtd^1~152^, Vtd^273~530^, Vtd^625~715^) can form a complex with full-length Mts (Supplementary Fig. S1c). These data confirm that Vtd is a binding partner of Mts, and Mts interacts with the conserved domains of Vtd.

### Mutations in *mts* and *vtd* synergistically result in mitotic defects

**Figure 2:**
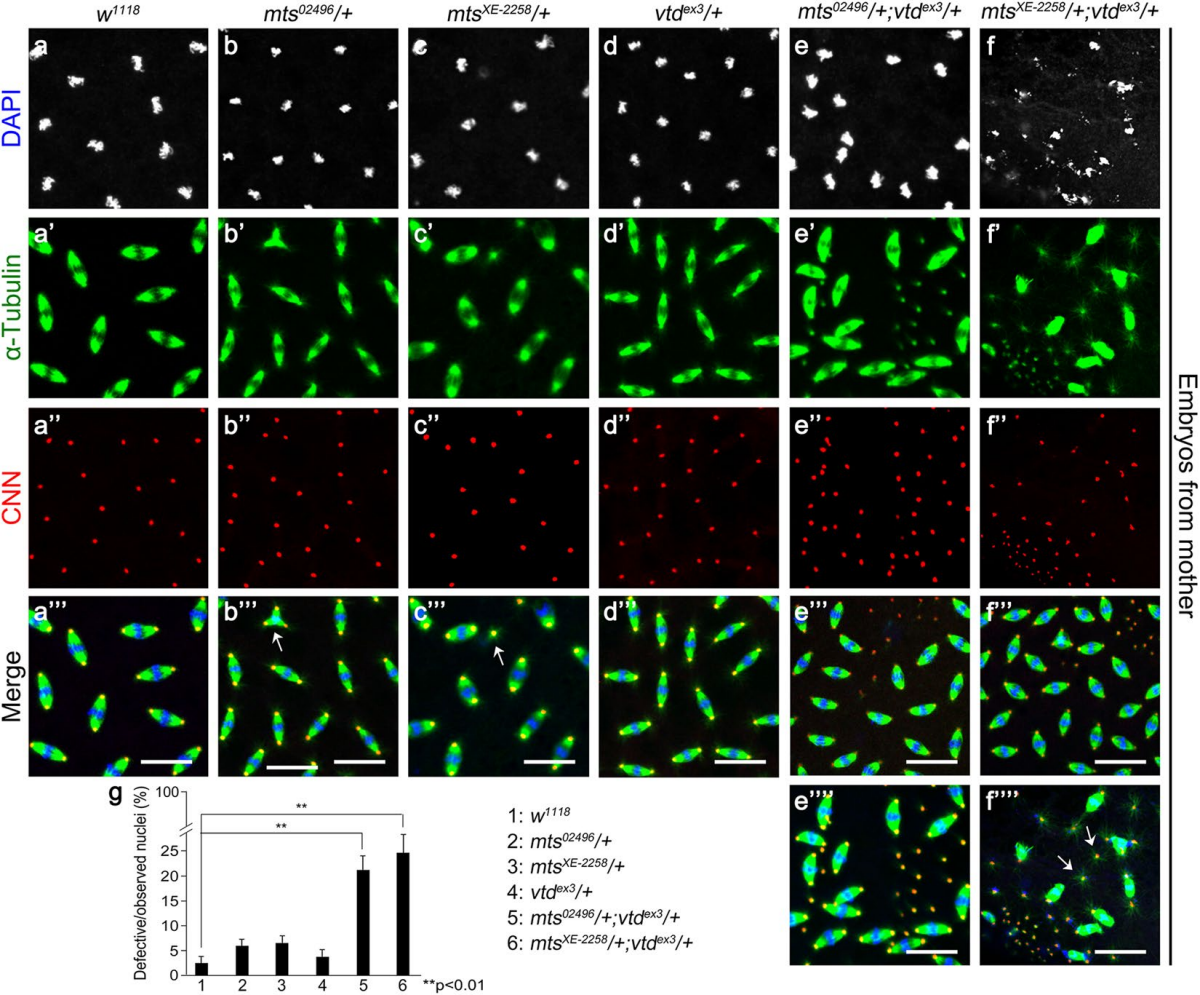
*mts* and *vtd* mutants show genetic interaction during mitosis in syncytial embryo. Embryos were collected from mother flies with the indicated genotypes and stained by DAPI (white), anti-CNN (red), and anti-Tubulin (green) antibodies. All embryos were approximately 2hr-old. (**a**) Control embryos from *w*^*1118*^ mothers show normal nuclei at metaphase during mitosis. Separate and merged channels are shown as indicated. (**b**,**c**) Embryos from *mts*^*02496*^/+ (**b**) and *mts*^*XE-2258*^/+ (**c**) mothers. Embryos from *mts* heterozygote mutant mothers show mild mitotic defects (arrows in **b”’**,**c”’**). (**d**) Embryos from *vtd*^*ex3*^/+ mothers are nearly normal. (**e**,**f**) Embryos from *mts*/+; *vtd*/+ double heterozygous mothers. ‘+’ indicates wild-type chromosomes instead of balancers. Both genotypes in (**e**,**f**) show severe defects in nuclear division. Free centrosomes often show star-shaped spindles (arrows). Scale bars, 20 µm. (**g**) Quantification of defective nuclei in syncytial embryos shown in (**a**) to (**f”’’**). The y-axis indicates percentage of all mitotic nuclei showing abnormal pattern of spindles and centrosomes. The number of free centrosomes were also included. Because it is not possible to determine the number of nuclei that provided free centrosomes, we arbitrarily scored each free centrosome as one abnormal mitotic nucleus. Therefore, the y-axis shows only approximate quantification of abnormal mitotic nuclei. Error bars are s.d. n = 10. **P < 0.01 (t-test).

To determine whether Mts and Vtd are functionally related in mitosis, we examined nuclear division cycles during early embryogenesis. Nuclear divisions in syncytial embryos are mainly regulated by maternal gene products. Therefore, we analyzed embryos obtained from crosses between *mts*/+ and/or *vtd*/+ heterozygous mutant females and wild-type males. To eliminate any effects of balancer chromosomes, *mts*/+ and *vtd*/+ females were generated from crosses between *mts/CyO* and *vtd/TM6b* heterozygotes and wild-type flies, respectively. During metaphase of the nuclear division cycles 9–13, embryos from *mts* heterozygous females (*mts*^*02496*^/+ or *mts*^*XE-2258*^/+) showed nearly normal pattern of mitosis. However, there were occasional abnormalities such as branched spindles or chromosome-free centrosomes at low frequencies (about 5% for both *mts* alleles), implying that these mutations have weak dominant effects in heterozygotes (Fig. 2b–b”’,c–c”’,g). Embryos from *vtd* heterozygous mutant females (*vtd*^*ex*^^3^/+) showed the normal pattern of mitosis without detectable dominant phenotypes (Fig. 2d–d”’,g).

In contrast with *mts*/+ or *vtd/*+ heterozygote, embryos from double heterozygous mutants for *mts* and *vtd* genes (*mts*^*02496*^/+; *vtd*^*ex3*^/+ or *mts*^*XE-2258*^/+; *vtd*^*ex3*^/+ without balancers) exhibited severe mitotic defects in about 17 and 20% of the nuclei scored, respectively (Fig. 2e–e””,f–f””). The term ‘nuclei’ is used to score defective mitosis in embryo even though most nuclear membranes are lost during mitosis, albeit not entirely^44^. For each genotype, about 1700 mitotic nuclei from 10 embryos were scored for quantification of different phenotypes (Fig. 2g). Most common defects in double heterozygotes for *vtd*^*ex3*^ and two different *mts* alleles were centrosomes without chromosomes and abnormally fused or branched spindle microtubules. Embryos from *mts*^*XE-2258*^/+; *vtd*^*ex3*^/+ heterozygotes showed higher frequency of monopolar spindles and free centrosomes with star-shaped spindles than *mts*^*02496*^/+; *vtd*^*ex3*^/+ heterozygotes. Such centrosomes with star-shaped spindles were similar to the “microtubule-star” phenotype seen in *mts* mutant embryos^8^. These genetic interactions between *mts* and *vtd* heterozygotes suggest that Mts and Vtd cooperate in the process of mitosis.

**Figure 3:**
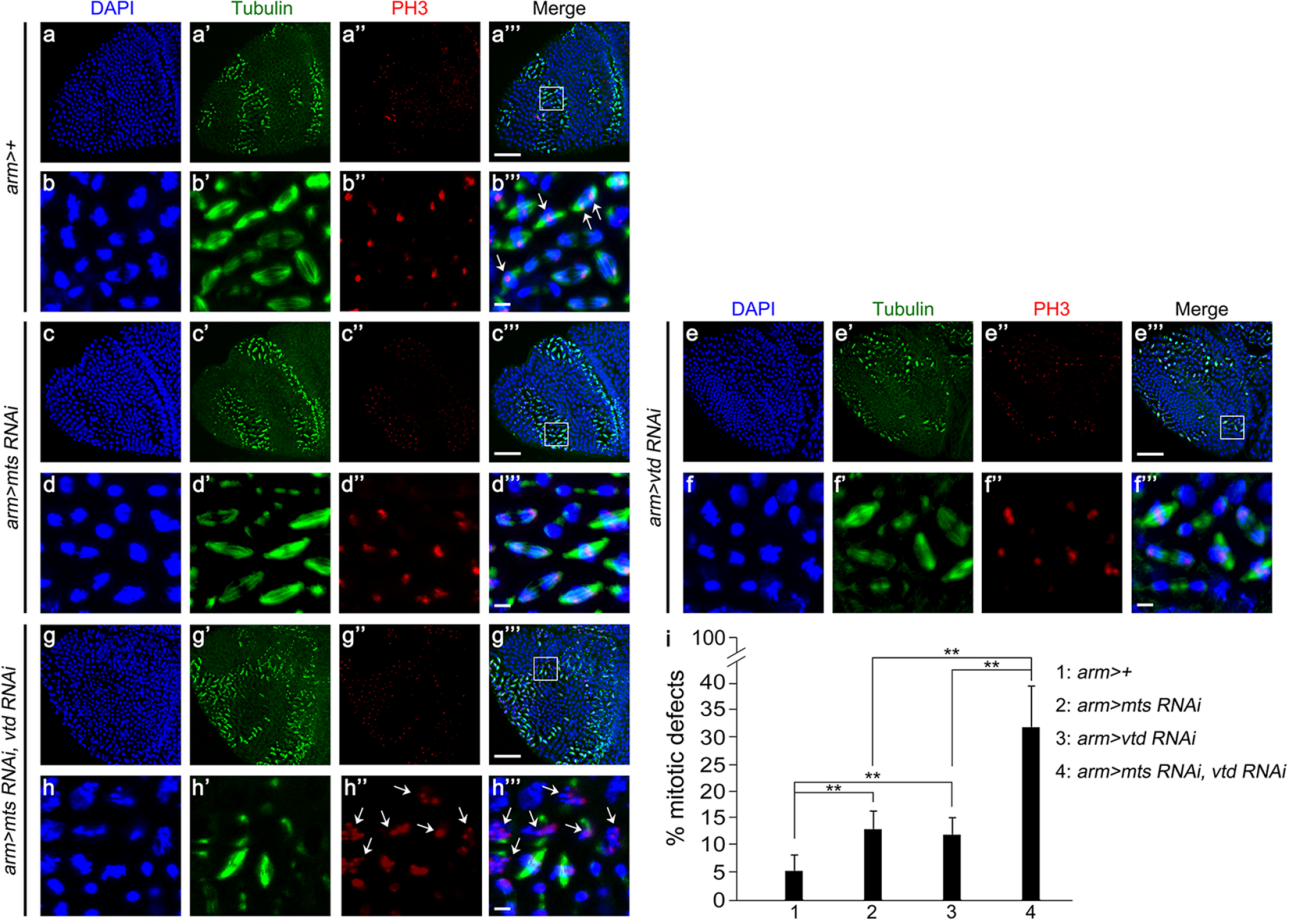
Double knockdown of *mts* and *vtd* causes synergistic mitotic defects in cellularized embryos. Approximately 4hr-old embryos were collected and stained by DAPI (blue), anti-Tubulin (green), and anti-PH3 (red) antibodies. Separate and merged channels are shown as indicated. (**a**,**c**,**e**,**g**) Low magnification views of mitotic domains in the head region (Scale bars, 20 µm). (**b**,**d**,**f**,**h**) Higher magnification views of the boxed areas in the mitotic domains (Scale bars, 10 µm). (**a**,**b**) *arm* > + control embryos. Note that the majority nuclei are at metaphase or anaphase. PH3 staining is normally detected at metaphase plate or near the pole at anaphase (arrows in **b”’**). (**c**,**d**) *arm* > *mts RNAi*. (**e**,**f**) *arm* > *vtd RNAi*. PH3 staining in (**d”**–**d’”**) and (**f”**–**f”’**) are similar to the pattern in the control embryo. (**g**,**h**) *arm* > *mts RNAi, vtd RNAi*. Double knockdown causes scattered PH3 puncta (arrows in **h”’**). (**i**) Quantification of mitotic defects in mitotic domains of cellularized embryos shown in (**a**) to (**h”’**). Nuclei showing any mitotic defects such as abnormal spindles and irregular distribution of PH3-labelled chromosomes were scored. The y-axis indicates the fraction of abnormal mitotic nuclei in percentage. Error bars are s.d. n = 8. **P < 0.01 (t-test).

In early embryogenesis, nuclei undergo mitotic divisions without cytokinesis to generate syncytium, a multinucleate single cell embryo. After 14th nuclear division cycle, syncytial embryo undergoes cellularization^45^. We examined whether *vtd* and *mts* also show mitotic defects in cellularized embryo. We induced *mts* RNAi and/or *vtd* RNAi ubiquitously in cellularized embryos by *armadillo (arm)-GAL4* (labelled as *arm* > *mts RNAi* or *arm* > *vtd RNAi*). Approximately 4h-old embryos were stained for tubulin and phosphohistone H3 (PH3), and examined for the pattern of mitosis in the region anterior to the cephalic furrow. The pattern of chromosome alignment and segregation was normal in *arm* > + control embryos. PH3-stained chromosomes were aligned at the equator in metaphase while segregating anaphase chromosomes were detected near the poles. Knockdown of *mts* (Fig. 3c–c”’,d–d”’,i) or *vtd* (Fig. 3e–e”’,f–f”’,i) using *arm-Gal4* resulted in similar defects in patterns of chromosomes and PH3 staining. In contrast, *arm* > *mts RNAi, vtd RNAi* double knockdown embryos showed severe mitotic defects, including abnormal spindle morphology and irregular distribution of PH3-labelled chromosomes (Fig. 3g–g”’,h–h”’,i). These data suggest that reduction of Mts and Vtd synergistically impairs chromosome alignment during metaphase possibly due to abnormal spindles and that genetic interaction between *mts* and *vtd* is important for cell division in cellularized embryos as well as in syncytial embryos.

### Reduced Mts and Vtd leads to loss of Cut expression and wing defects

**Figure 4:**
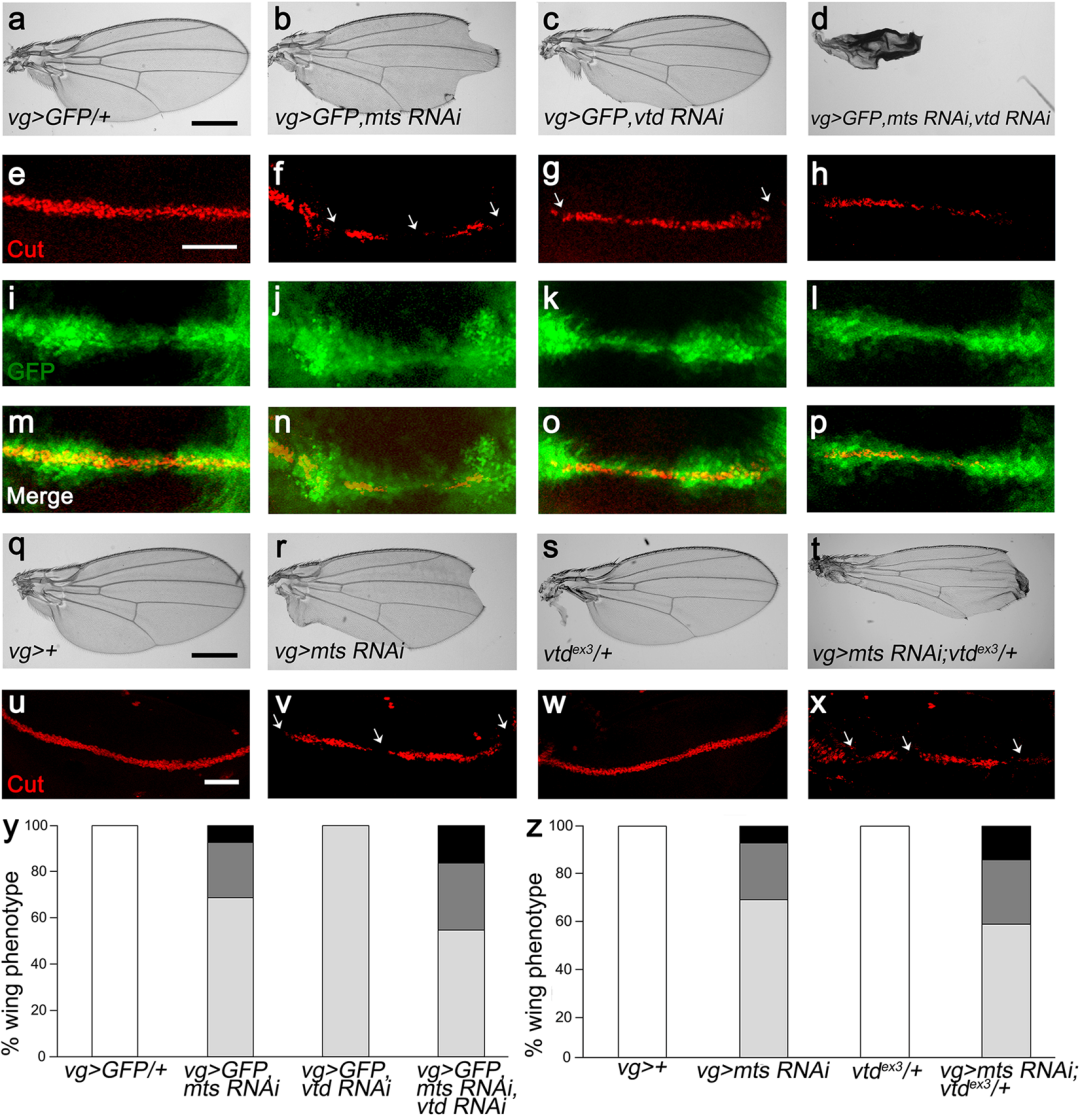
Vtd reduction enhances notching phenotypes in *mts* knockdown wing. Effects of RNAi knockdown of *mts* and/or *vtd* driven by *vg-Gal4* in the wing. (**a**–**d**) Female adult wings of indicated genotypes. Double knockdown of *mts* and *vtd* shows severe wing reduction (**d**). Scale bar, 100 µm. (**e**–**h**) Cut staining in wing imaginal disc from 3^rd^ instar larvae. Cut staining is strongly reduced or lost at the DV boundary of wing imaginal disc (white arrows). Scale bar, 50 µm. (**i**–**l**) GFP expression by *vg-Gal4* show the presence of DV boundary cells. (**m**–**p**) Merges of (**e**–**l**). (**g**–**t**) Female adult wings of indicated genotypes. *mts* knockdown in *vtd* heterozygote (**t**) enhances the notching phenotype by *mts* RNAi (**r**). (**u**–**x**) Cut staining of wing imaginal discs of the genotypes shown in (**q**–**t**). Scale bar, 50 µm. (**y**,**z**) Quantification of wing phenotypes. White: normal wing, Light grey: mild wing notching (less than 40% reduction in wing size), Dark grey: severe wing notching (41–70% reduction in wing size), Black: near complete wing loss (more than 71% reduction in wing size) (**y**) Quantification of adult wing phenotypes in (**a**–**d**). (**z**) Quantification of adult wing phenotypes in (**q**–**t**).

The Cohesin complex is also known to regulate gene expression. Cohesin binds to a region between a remote wing margin enhancer and the promoter of the *cut* locus^38^. The *cut* gene encodes a homeodomain protein expressed along the boundary between dorsal and ventral compartments in wing disc. It has been proposed that cohesin inhibits *cut* expression based on the fact that wing notching phenotype caused by a *cut* mutant allele *ct*^*k*^ is partially suppressed by *Rad21* RNAi^38^. To see whether *mts* and *vtd* show genetic interaction in Cut expression, we examined both wing discs and adult wings. *mts* knockdown in the DV boundary region by *vg-Gal4* (*vg* > *GFP, mts RNAi*) resulted in large notches in the wing margin (Fig. 4b,y). Because the DV boundary of wing disc develops to the margin of an adult wing, this notching phenotype suggests a role of Mts in wing margin growth. Unexpectedly, however, Vtd depletion in the wing DV boundary (*vg* > *GFP, vtd RNAi*) also caused wing notching, although the phenotype was milder than that of *vg* > *mts RNAi* (Fig. 4c,y). Two independent *vtd* RNAi lines showed notching phenotypes in adult wings. Strikingly, double knockdown of *mts* and *vtd* (*vg* > *GFP, mts RNAi, vtd RNAi*) showed severe growth defect, leading to a loss of nearly 90% of wing tissue (Fig. 4d,y). Since *vtd* RNAi has only weak effects on wing development, the strong wing phenotype by double knockdown are likely due to a synergistic genetic interaction between *mts* and *vtd*.

Expression of the *cut* gene at the DV boundary is essential for wing margin development^46^. We checked the Cut level in 3rd instar wing disc (Fig. 4e~h) and found partial loss of Cut in *vg* > *GFP, mts RNAi* wing disc (Fig. 4f). Cut expression was also reduced in wing discs from *vg* > *GFP, vtd RNAi* (Fig. 4g), consistent with the result that *vtd* RNAi causes mild notching in adult wings (Fig. 4c). Wing discs with double knockdown of *mts* and *vtd* showed stronger reduction in the Cut level than *mts* or *vtd* single knockdown (Fig. 4h). We also checked the pattern of GFP expression in the DV boundary region driven by *vg-GAL4*. In all genotypes tested, GFP was expressed similarly along the DV boundary (Fig. 4i~l). Therefore, loss of Cut resulting from the knockdown of *mts* and/or *vtd* was not due to cell death at this larval stage.

In addition to *vtd* RNAi, we tested whether *vtd* mutation can enhance the wing phenotype caused by *mts* RNAi knockdown. Homozygous *vtd*^*ex3*^ mutant die during embryogenesis^43^, but *vtd* heterozygous mutation (*vtd*^*ex3*^/+) did not affect wing development (Fig. 4s,z). However, *vtd*^*ex3*^ heterozygotes enhanced the moderate wing phenotype of *vg* > *mts RNAi*, resulting in a stronger reduction of the wing size (Fig. 4t,z). Likewise, *vtd* heterozygote (*vtd*^*ex3*^/+) showed normal Cut level in wing disc (Fig. 4w) but strongly enhanced the partial loss of Cut expression caused by *mts* RNAi (Fig. 4x). These results suggest that genetic interaction between Mts and Vtd is required for normal Cut expression and wing development. Because *mts* RNAi phenotype was enhanced by reducing Vtd, we examined whether Vtd overexpression may be sufficient to suppress the effects of *mts* RNAi. However, Vtd overexpression, which was confirmed by immunostaining (Supplementary Fig. S2e–e”’), could not affect the *mts* RNAi phenotype (Supplementary Fig. S2a–d).

Taken together, Mts and Vtd are essential for development of wing imaginal discs and show genetic interaction, as in mitosis during embryogenesis.

### Loss of Mts or Tws causes reduction of Vtd level

**Figure 5:**
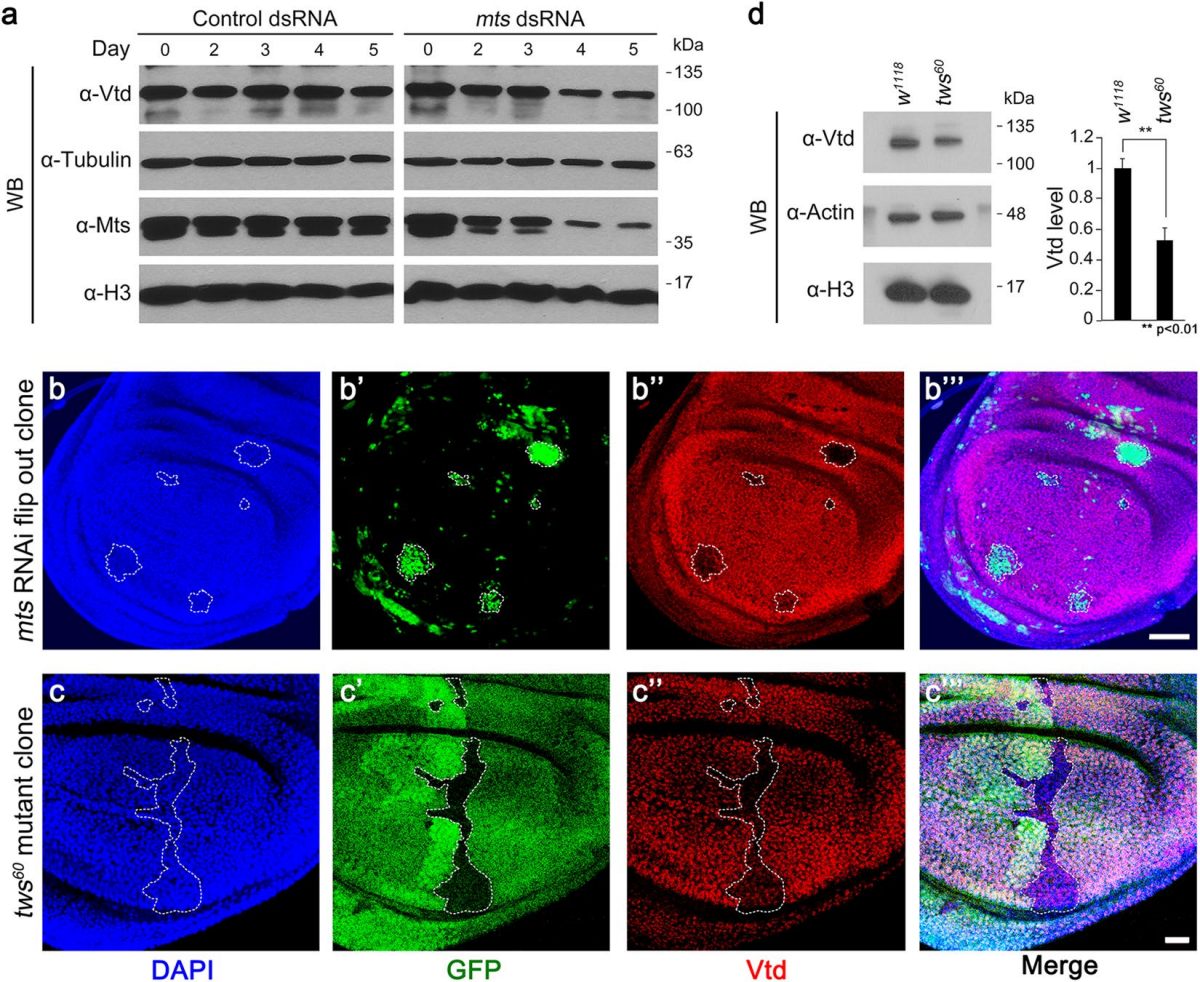
*mts* knockdown causes reduction of Vtd level in S2 cells and wing imaginal disc. (**a**) Immunoblot using extracts from S2 cells treated with control dsRNA (*pSK* vector) or *mts* dsRNA. Mts level is significantly reduced from Day 4. Vtd level is also reduced from Day 4. Immunoblot with anti-Tubulin and anti-Histone H3 staining is used as controls to compare the amount of Mts or Vtd. (**b**–**b”’**,**c**–**c”’**) Wing imaginal discs were stained by DAPI (blue), anti-Vtd (red) antibodies. (**b**–**b”’**) GFP^+^ cells are *mts* knockdown clones induced by *hs-FLP*; *Ay* > *mts RNAi*. *mts* RNAi clones show reduction of Vtd. Scale bar, 20 µm. (**c**–**c”’**) *tws*^*60*^ mutant clones marked by the lack of GFP expression show reduction of Vtd staining. Scale bar, 20 µm. (**d**) Immunoblot using extract from *w*^*1118*^ wing imaginal disc (control) or *tws*^*60*^ homozygous wing imaginal disc (left panel). Quantitative analysis (right panel). Vtd levels are normalized to the amount of Histone H3. Error bars are s.d. n = 3. **P < 0.01 (t-test). (**a**,**d**) Full-length blots are presented in Supplementary Fig. 6.

Our data thus far suggest that Mts and Vtd are functionally related. Since *mts* RNAi phenotype in the wing is enhanced by reducing the Vtd level but cannot be rescued by Vtd overexpression, we hypothesized that Mts might be necessary for the Vtd function. To test this possibility, we knocked down Mts in S2 cells using *mts* dsRNA and examined the level of Vtd (Fig. 5a). Western blot analysis showed that Mts level begins to decrease from Day 2 after *mts* dsRNA treatment and is significantly reduced from Day 4. Interestingly, Vtd level was also reduced from Day 4 (Fig. 5a), indicating that Mts is required to maintain the level of Vtd protein. To confirm this result *in vivo*, we examined the effects of depleting Mts in wing discs. We generated clones of cells expressing *mts* RNAi by using the flip-out method in *Ay-Gal4 (Act5C* > *y*^+^ > *GAL4)* wing discs (Fig. 5b–b”’). *mts* RNAi clones were induced by heatshock flippase (hs-FLP). As shown in Fig. 5b–b”’, *mts* RNAi clones marked by GFP expression showed significant reduction in the Vtd level.

To support the role of Mts for the regulation of Vtd level, we tried to examine *mts* loss-of-function mutant clones in wing discs using the FLP-FRT system^47^. However, *mts* mutant clones were not formed due to cell lethality or were too small to analyze the mutant effects (data not shown). As an alternative approach, we utilized a mutation in *twins* that encodes a regulatory subunit of PP2A. Unlike *mts* mutant clones, large *tws*^*60*^ mutant clones were recovered in wing discs using *hs-FLP* (Fig. 5c–c”’). Consistent with the results from *mts* RNAi clones, the level of Vtd expression was strongly reduced in *tws*^*60*^ mutant clones. We also measured the effects of *tws*^*60*^ mutation by western blot analysis of cell extracts from wing discs. Levels of Vtd in *tws*^*60*^ mutant wing discs were reduced to about 50% compared with the wild-type discs (Fig. 5d), while there was no change in the level of control proteins like actin and histone H3. To determine whether Mts affects *vtd* transcription, we checked *vtd* mRNA level in *tws*^*60*^ homozygous wing discs (Supplementary Fig. S3). Quantitative PCR results showed that the relative amount of *vtd* mRNA was not significantly changed in *tws*^*60*^ homozygous wing discs. These data indicate that PP2A is required mainly to maintain the level of Vtd protein but not *vtd* mRNA expression.

Based on the effects of *tws*^*60*^ allele on the Vtd level (Fig. 5c–c”’), it was possible that Tws might also form a complex with Vtd. Indeed, 2xMyc-tagged-Vtd and V5-tagged-Tws showed co-immunoprecipitation (Supplementary Fig. S4a). Furthermore, when both V5-tagged Vtd and V5-tagged Tws were transfected to S2 cells with 2xFLAG-tagged Mts, FLAG-Mts coimmunoprecipitated with V5-Tws and V5-Vtd (Supplementary Fig. S4b). These results suggest that Mts interacts with Tws and Vtd.

### Vtd stability is regulated by PP2A through proteasomal degradation pathway

**Figure 6:**
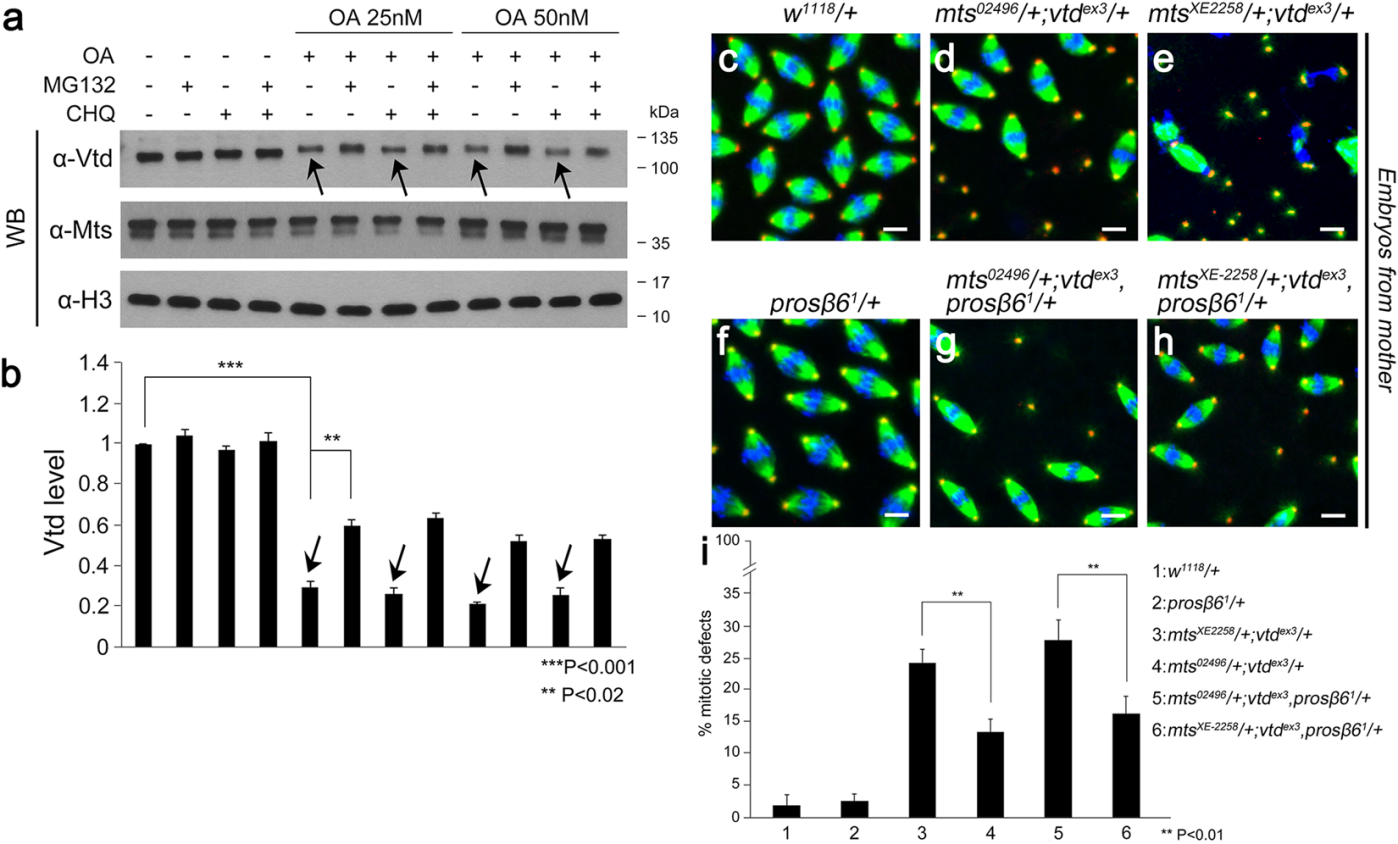
Vtd stability is regulated by PP2A through proteasomal degradation pathway. (**a**) Effects of protein degradation inhibitors on the level of Vtd and Mts. Immunoblot of extracts from S2 cells treated with Okadaic acid (OA), MG132 and Chloroquine (CHQ) as indicated. OA was used at 25 nM and 50 nM. MG132 and CHQ were used at 100 µM and 500 µM, respectively. Note that OA treatments decrease the level of Vtd in the absence of MG132 (arrows). Full-length blots are presented in Supplementary Fig. 6. (**b**) Quantitative analysis of Vtd levels shown in the Western blot in (**a**). Vtd levels are normalized to the level of Histone H3 and the control level of Vtd in the absence of OA, MG132 and CHQ. Error bars are s.d. n = 3. **P < 0.01, ***P < 0.001 (t-test). (**c**–**i**) Embryos from mother flies of the indicated genotypes. Approximately 2 hr-old embryos were stained with DAPI (white), anti-CNN (red), and anti-Tubulin (green) antibodies. Embryos from double heterozygotes for *mts* and *vtd* show severe defects with frequent free centrosomes (**d**,**e**) and chromosomal disruption (**e**). Reduction of proteasome function by *prosβ*6^*1*^/+ significantly suppresses the *mts*/+; *vtd*/+ phenotypes (**g**,**h**). ‘+’ indicates wild-type chromosomes rather than balancers. Scale bars, 10 µm. (**i**) Quantification of embryo phenotypes. ‘% mitotic defects’ on the y-axis indicate percentage of all mitotic nuclei showing abnormal pattern of spindles. The number of free centrosomes were also included, as explained in Fig. 1 legend. Error bars are s.d. n = 10. **P < 0.01 (t-test).

The results from *mts* knockdown in S2 cells and clonal analysis in wing discs strongly suggest that PP2A regulates Vtd stability. Since *mts* knockdown reduces the Vtd level, we tested whether Vtd stability is regulated by a protein degradation pathway. When S2 cells were treated with okadaic acid to inhibit PP2A activity^48^ (Fig. 6a,b), Vtd level was significantly decreased. This result supports that PP2A activity is required to regulate Vtd stability. There are two major protein degradation pathways in eukaryotic cells, that is, the ubiquitin-proteasomal pathway and the lysosomal degradation pathway^49–51^. To distinguish these possibilities, we treated S2 cells with chloroquine (500 µM, inhibitor of lysosome) and/or MG132 (100 µM, inhibitor of proteasome) in the presence or absence of okadaic acid (Fig. 6a,b). The reduction of Vtd level by okadaic acid was not recovered by chloroquine. On the contrary, the effect of okadaic acid on the Vtd level was significantly recovered in S2 cells co-treated with MG132. These data support that PP2A regulates Vtd stability through the proteasomal degradation pathway.

To confirm that Vtd stability is regulated *in vivo* by the proteasomal degradation pathway, we checked whether proteasome inhibition can rescue the mitotic defects in embryos laid from *mts*/+; *vtd*/+ double heterozygous mutants (Fig. 2e–e”’’,f–f”’’, 6d,e,i, Supplementary Fig. S5b–b”’,c–c”’). We used *prosβ*6^*1*^ allele^52,53^, a dominant mutation in the Prosβ6 subunit of proteasome. Early embryos from *mts*/+; *vtd*/+ double heterozygous mutants showed severe mitotic defects (Fig. 6d,e,i, Supplementary Fig. S5b–b”’,c–c”’), but the *prosβ*6^*1*^/+ heterozygote condition partially reduced mitotic defects by approximately two-fold in these embryos from *mt*s/+; *vtd*/+ mothers (Fig. 6g,h,i, Supplementary Fig. S5e–e”’,f–f”’). The effects of the *prosβ*6^*1*^ mutation were similar for two double heterozygotes with different *mts* alleles (*mts*^*02496*^/+; *vtd*^*ex3*^/+ or *mts*^*XE-2258*^/+; *vtd*^*ex3*^/+). These results suggest that Vtd stability is regulated by PP2A through the proteasomal degradation pathway.

## Discussion

We have shown that Mts and Vtd are required for normal mitosis during embryogenesis and Cut regulation during wing development. Cohesin is necessary for sister chromatid cohesion, but it is also involved in the organization of spindle poles and microtubules in the division process, although the underlying mechanism is not well understood^21^. It has been shown that centrosomes in *mts* mutant embryos can nucleate microtubules but the growing microtubules are rarely associated with chromosomes^8^. Similarly, embryos double heterozygous for *mts* and *vtd* mutations show chromosome-free centrosomes with abnormal spindles (Fig. 2). It is possible that defects in spindle formation or its connection to the chromosomes might lead to falling of associated chromosomes into the interior of embryo, resulting in free centrosomes. It has been shown that human cells depleted in Rad21 show defects in centrosome integrity^54^. Hence, it is plausible that free centrosome phenotypes might also be related to abnormalities in centrosomes.

We have also shown that Mts and Vtd are required for the maintenance of Cut level in wing disc for normal wing development. Importantly, knockdown of *mts* and *vtd* in wing discs results in synergistic effects to disrupt wing development. It has been reported that cohesin acts as an insulator to prevent the long-range communication between promoter and enhancer of the *cut* gene, thereby inhibiting Cut expression^37,38^. This idea is based on the findings that knockdown of SA or Rad21 cohesin subunit partially suppresses the wing notching phenotype caused by *ct*^*k*^ mutation. *ct*^*k*^ is a gypsy transposon insertion allele that partially inhibits an enhancer-promoter interaction for *cut* expression. The Scc2 homolog Nipped-B interacts with the cohesin complex to regulate gene expression as well as sister chromatid cohesion. Nipped-B is an adherin that loads the cohesin complex onto chromosomes. Cohesin is loaded from the later stage of anaphase till prophase, and therefore it is a structural component of interphase chromosomes where gene expression occurs^38^. In contrast to SA, Nipped-B acts to promote *cut* expression. Our data show that *vtd* knockdown causes reduction of Cut level at the DV boundary in wing disc, resulting in notched wings. This result is in contrast to the earlier findings that Rad21 functions as a negative regulator of *cut* expression.

Although the basis for this discrepancy is not understood, it is worth noting that there are several experimental differences between the previous studies^37,38^ and this study. In the previous studies, the relationship between Rad21 and Cut expression was determined by examining whether wing notching phenotype of *ct*^*k*^ mutant can be modified by *Rad21* RNAi. Since this assay is based on the modification of *ct*^*k*^ phenotype rather than the level of cut expression *per se*, it is unknown whether this phenotypic modification is correlated with the changes in the *cut* expression. In contrast, our approach in this study was to directly examine the effects of *vtd* RNAi on the Cut protein level along the DV boundary and on wing notching. In addition, previous studies induced RNAi using the Sym-pUAS vector to generate dsRNA by bidirectional mRNA synthesis whereas we used a TRiP line which expresses short hairpin (sh)RNA instead of long dsRNA^55^. Use of different RNAi methods may not significantly affect *vtd* knockdown effects. However, differences in the Gal4 drivers used to express *Rad21* RNAi might be important. Previous studies used *heatshock70* (*hsp70*)-*Gal4*, thus inducing *Rad21* RNAi ubiquitously while *vtd* RNAi in this work was targeted more specifically to the DV boundary of wing disc by *vg-Gal4*. Interestingly, it was observed in the previous studies that *Rad21* RNAi increases *SA* mRNA and *Rad21* mRNA in some cases. Thus, potential cross-regulation interactions between cohesin subunit mRNA levels might also influence the effects of *Rad21* RNAi in the *ct*^*k*^ background. Nonetheless, our direct observation of the reduced Cut expression in the DV boundary of wing disc and wing notching by *vtd* RNAi is in accord with the positive role of Vtd in *cut* regulation, which is also consistent with the Nipped-B requirement for *cut* expression. Since reduced Mts strongly enhances the notching phenotype of *vtd* mutation or RNAi, Mts appears to be critical for promoting the Vtd function in *cut* gene activation. This is supported from the reduced Cut expression at the DV boundary by double knockdown of *mts* and *vtd*.

As a mechanism underlying the relationship between Mts and Vtd, we provided evidence that *mts* knockdown or PP2A inhibition by okadaic acid causes a reduction in the Vtd protein level in S2 cells. In addition, *mts* RNAi or loss of Tws in mutant clones in wing discs significantly reduced the level of Vtd *in vivo*. Furthermore, our data indicate that Mts is required for preventing the proteasome-dependent degradation of Vtd *in vivo*. Thus, we propose that phosphorylation of Vtd causes its downregulation by the proteasome pathway. In this process, PP2A is critical to maintain the level of Vtd by antagonizing Vtd phosphorylation. The role of PP2A in the regulation of Vtd stability is required in both embryonic mitosis and wing development based on (i) the reduction of Vtd by depleting Mts or Tws in wing discs and (ii) the suppression of mitotic defects from *mts/vtd* double knockdown by reducing the proteasome function in embryos.

Our biochemical data show that the C-terminal domain of Mts binds to Vtd, suggesting that PP2A might be involved in dephosphorylation of Vtd. Since Mts physically interacts with Vtd, Vtd might be a direct substrate for the PP2A activity. However, we do not exclude the possibility that PP2A dephosphorylates other factors to regulate Vtd stability. Rad21 in yeast is phosphorylated by Polo kinase during mitosis^16,17^. Despite the similarities in protein sequences of Vtd and Rad21, Polo phosphorylation sites of Rad21 are not conserved in Vtd. This raises a possibility that cleavage of Vtd might be triggered by a different protein kinase(s) or through an unidentified mechanism. In S2 cells, phosphorylation of the SA subunit has been detected. A histone chaperone NAP1 involved in sister chromatid resolution is known to antagonize PP2A to prevent SA dephosphorylation^42^. Thus, it is possible that Mts-Vtd binding might affect the Mts-SA interaction. Identification of *in vivo* substrates for PP2A and new factors associated with the Mts-Vtd complex will help to reveal the mechanism of Vtd regulation. It has been reported that Twins (Tws), a regulatory subunit of PP2A, is required for Wingless (Wg)/Wnt signaling by stabilizing Arm during wing development^56^. However, it is unknown whether the control of Arm stability by Tws is related to the PP2A-dependent Vtd regulation. PP2A and cohesin complexes are evolutionarily conserved in their structures. It would be interesting to see whether the catalytic subunit of PP2A is also involved in stabilizing the Rad21/Scc1 subunit for mitosis and gene regulation in mammalian systems.

## Materials and Methods

### Fly stocks and genetics

All *Drosophila* strains were grown and maintained at room temperature. *w*^*1118*^ flies were used as the wild-type. *vg*-*GAL4* was a gift from Masayuki Miura. *arm-GAL4-arm-GAL4::UAS-a-Cat:eGFP/arm-GAL4::UAS-a-Cat:eGFP;UAS-Dcr2T10/UASDcr2T10* was from Ingrid Lohmann. *yw, hs-Flp; act5c* > *CD2* > *GAL4, UAS-GFP/TM6b* was from Daniela Grifoni. *tws*^*60*^ was provided by Tadashi Uemura. *UAS-vtd*^*WT*^*-myc*_*10*_ (This study). The following stocks were from the Bloomington Stock Center (Bloomington, USA), Vienna *Drosophila* Stock Center (Vienna, Austria): *UAS*-*mts* RNAi (Bloomington 27723, VDRC 41924), *UAS*-*vtd* RNAi (Bloomington 36786, VDRC 13669), *vtd*^*ex3*^ (Bloomington 27609), *mts*^*02496*^ (Bloomington 11193), *mts*^*XE-2258*^ (Bloomington 5684), *prosβ6*^*1*^ (Bloomington 6182), *vtd*^*ex3*^*,tub::Vtd*^*WT*^-myc_*10*_ (Bloomington 27615).

### Yeast two-hybrid screen

A cDNA fragment encoding the C-terminal Mts bait (67aa) was cloned into EcoRI/BamHI sites of pGBKT vector. This pGBKT-Mts-C-terminal vector was used for transformation of Y2H Gold yeast strain for bait. Mate & Plate™ Library - Universal *Drosophila* (Normalized) (Cat. No. 630485, Clontech, CA) was used for cDNA library. Yeast two hybrid screening and confirmation of positive interaction was performed according to the manufacturer’s instruction.

### Generation of anti-Vtd antibody

An antibody against Vtd was raised in rabbits with His-Vtd^452~674^ (His tagged amino acids 453–674) expressed in *Escherichia coli* by isopropyl β-D-1-thiogalactopyranoside induction, and the antibody was affinity-purified with GST-Vtd^452~674^ (GST tagged amino acids 453–674). Antibody production and purification were carried out by Younginfrontier (Seoul, Korea). Purified Vtd antibody was used for immunoblotting (1:5000) and for immunohistochemistry (1:500).

### Immunohistochemistry

Embryo fixation was performed according to standard methods. Embryos were fixed by a solution containing heptane (Sigma, St Louis, MO) and methanol^57,58^. Wing discs were fixed in PLP fixative (2% paraformaldehyde, 75 mM lysine, and 35 mM phosphate buffer, pH7.4) for 15–30 min at room temperature.

Antibodies used for immunohistochemistry were as follows: mouse anti-Cut (1:200, 2B10, Developmental Studies Hybridoma Bank (DSHB), Iowa City, Iowa), guinea pig anti-Centrosomin (1:1000, from Jordan Raff), Rat anti-α-Tubulin (1:200, MAB1864, Millipore, Burlington, Massachusetts), rabbit anti-PH3 (1:200, 06–570, Millipore), Rabbit anti-Vtd (1:500, this study), rabbit anti-Myc (1:100, ab9106, Abcam, Cambridge, UK). Secondary antibodies conjugated with Rhodamine Red™-X (RRX), Alexa Fluor® 647 or fluorescein isothiocyanate (1:200, 715-095-151, 715-295-151, 715-605-151, 711-095-152, 711-295-152, 711-605-152, 706-095-148, 706-295-148, 706-605-148, 712-095-153, 712-295-153, 712-605-153) were from Jackson ImmunoResearch Inc. Vectashield with 4′, 6-diamidino-2-phenylindole (H-1200, Vector Laboratories, Burlingame, CA) was used for mounting. Fluorescent images were acquired using Carl Zeiss LSM710 confocal microscope (Carl Zeiss, Oberkochen, Germany).

### *In vitro* GST-pull down assay

For GST pull-down, R2 cells (BL21 derivative) were transformed with plasmids for MBP–Mts^WT^ and GST–Vtd^WT^. Pull-down buffer (PDB) contained 20 mM Tris pH 7.5, 150 mM NaCl, 0.5 mM EDTA, 10% glycerol, 0.1% Triton X-100, 1 mM dithiothreitol and protease inhibitor cocktail. Five micrograms of MBP fusion proteins and GST fusion proteins were used as prey and baits, respectively. For western blotting, Mouse anti-MBP (1:20000, E8032S, NEB, Ipswich, Massachusetts), Mouse anti-GST (1:4000, sc-138, Santa Cruz, Dallas, Texas), and secondary antibody with HRP (1:20000, 715-035-151, 711-035-152, Jackson ImmunoResearch Laboratories, West Grove, PA) were used.

### Cell culture and transfection

*Drosophila* S2 cells were cultured in M3 media (Sigma, Saint Louis, Missouri) with 10% Fetal Bovine Serum (Thermo, Waltham, MA). S2 cells were transfected using Effectene (Qiagen, Venlo, Netherlands) or X-tremeGENE HP DNA transfection reagent (Roche, Basel, Switzerland) according to the manufacturer’s manual. pAc5.1 empty vector was used to transfect with an equal amount of plasmids. A total of 1–3 μg DNA was used for each transfection. For co-IP, transfected S2 cells were incubated during 2~3 day for producing proteins from transfected DNA.

### Co-immunoprecipitation (co-IP) and Western blotting

For co-IP, cell extracts were obtained from S2 cells or embryos. Embryos were collected at different stages as indicated. 20 wing discs were used for checking Vtd level by Western blotting. S2 cells, embryos and wing discs were lysed on ice in cold IP buffer (20 mM HEPES (pH 7.4), 0.2 mM EDTA, 1.5 mM MgCl_2_, 1 mM DTT, 5% glycerol, 80 mM KCl, 0.2% NP-40, 1x proteinase inhibitor cocktail and 1x phostop (Roche)). Protein complexes were immunoprecipitated with 1~2 µg antibody at 4 °C for 2 h. Antibody-protein complexes were immunoprecipitated by protein G agarose beads (Roche) or SureBeads™ Protein G Magnetic Beads (Biorad, Hercules, California). The samples were boiled in protein loading buffer at 94 °C for 5 min and loaded for SDS-PAGE and western blotting.

The following antibodies were used for IP: Mouse anti-V5 (R960-25, Invitrogen, Waltham, MA), Rabbit anti-FLAG (V8137, Sigma) and Mouse anti-FLAG (F1804, Sigma), Rabbit anti-Myc (ab9106, Abcam), Rabbit IgG (I-1000, Vector); Antibody for western blotting: Mouse anti-V5 (1:5000, R960-25, Invitrogen), Rabbit anti-FLAG (1:2500, V8137, Sigma), Mouse anti-FLAG (1:2000, F1804, Sigma), Rabbit anti-Vtd (1:5000, this study), Mouse anti-β-Tubulin (1:10000, E7, DSHB), Mouse anti-Mts (1:5000, 610555, BD Transduction Laboratories™, San Jose, CA), Rabbit anti-Histone H3 (1:10000, 05–928, Millipore), Mouse anti-Actin (1:10000, ab8224, Abcam), Secondary antibodies (1:20000, 715-035-151, 711-035-152, Jackson ImmunoResearch Laboratories).

### Double-stranded RNA-mediated interference

One microgram of PCR products with T7 promoter sequence (*pSK(*−*)*, *mts*) was used for production of double-stranded RNA using MEGAscript kit (Invitrogen). The bathing way was used for dsRNA treatment^59^. For knockdown of *mts*, S2 cells were treated with 30 µg of double-stranded RNA for target gene.

Primer sets for dsRNA

pSK(−) dsRNA Forward: taa tac gac tca cta tag g atc gat aag ctt gat atc gaa ttc

pSK(−) dsRNA Reverse: taa tac gac tca cta tag g gca ccg cct aca tac ctc gct

*mts* dsRNA Forward: taa tac gac tca cta tag g atg gag gat aaa gca aca aca aaa

*mts* dsRNA Reverse: taa tac gac tca cta tag g gta cac ctg tgt gat ctg gc

### Quantitative PCR (QPCR)

Wing discs from *tws*^*60*^ homozygous samples were frozen in liquid nitrogen and stored at −80 °C until use. Total RNA from wing discs was prepared with Trizol reagent (Invitrogen) according to the manufacturer’s instruction. Total RNA (2 µg) was reverse-transcribed using the QuantiTect reverse transcription Kit (Qiagen). QPCR was performed using SYBR Green master mix on CFX96 Real-Time PCR System (BioRad) with standard cycling parameters (1 min at 95 °C, 40 cycles of 20 sec at 95 °C, 20 sec at 58 °C, and 45 sec at 72 °C). C_T_ values for detected mRNA levels of each gene were normalized to those of *rp49*. Mean expression levels were calculated from the values of three independent experiments, and were indicated as fold-changes.

Primer sets used for QPCR

*rp49* Forward: aga tcg tga aga agc gca cca ag

*rp49* Reverse: cac cag gaa ctt ctt gaa tcc gg

*vtd* Forward: cag aaa aca ggc ggc aca aa

*vtd* Reverse: ggg ttt tcg aac gtt ggt cc

## Supplementary information

Drosophila PP2A - Kim et al Supplementary Information
