# Supplementary material for: Protein phosphatase 2A interacts with Verthandi/Rad21 to regulate mitosis and organ development in Drosophila
Source: Sci Rep. 2019 May 20;9:7624. doi: 10.1038/s41598-019-44027-3 (PMC6527568; doi:10.1038/s41598-019-44027-3)
Supplement: Supplementary file 1 — Drosophila PP2A - Kim et al Supplementary Information [file 41598_2019_44027_MOESM1_ESM.pdf]

Supplementary Information

Protein phosphatase 2A interacts with Verthandi/Rad21 to regulate mitosis and organ development in *Drosophila*

Authors

Lee-Hyang Kim[1], Sung-Tae Hong[2,3] and Kwang-Wook Choi[1*]

Affiliations

[1]Department of Biological Sciences, Korea Advanced Institute of Science and Technology (KAIST), Daejeon, Republic of Korea

[2]Department of Anatomy & Cell Biology, College of Medicine, Chungnam National University, Daejeon, Republic of Korea

[3]Department of Medical Science, College of Medicine, Chungnam National University, Daejeon, Republic of Korea

*Correspondence author: kchoi100@kaist.ac.kr (K.-W. C.)

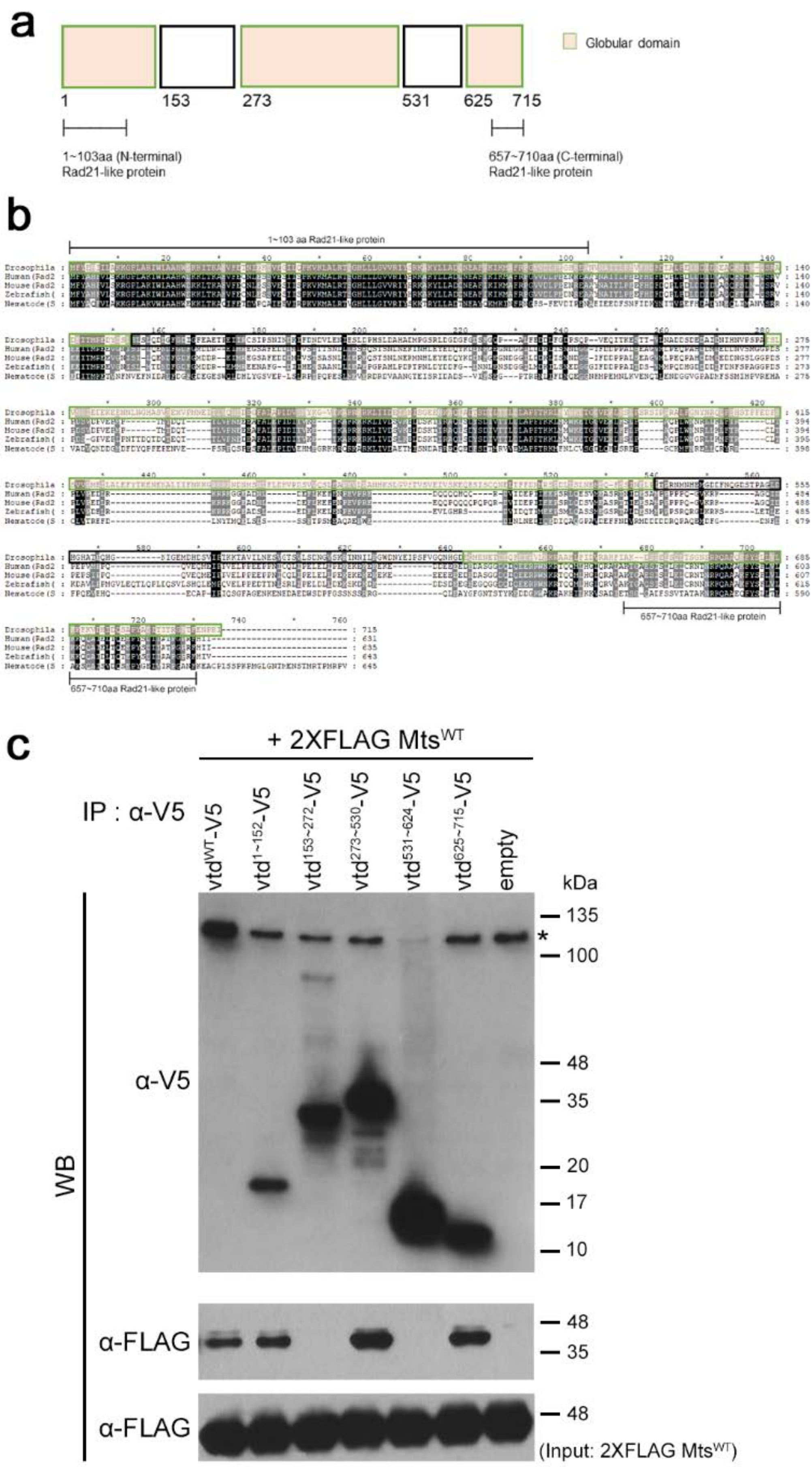
a
Globular domain
1
153
273
531
625
715
1~103aa (N-terminal)
Rad21-like protein
657~710aa (C-terminal)
Rad21-like protein
b
1~103 aa Rad21-like protein
Drosophila
Human (Rad2
Mouse (Rad2
Zebrafish (
Nematode (S
657~710aa Rad21-like protein
c
+ 2XFLAG Mts^WT
IP : α-V5
vtd^WT_V5
vtd^1~152_V5
vtd^153~272_V5
vtd^273~530_V5
vtd^531~624_V5
vtd^625~715_V5
empty
kDa
135
100
48
35
20
17
10
*
α-V5
WB
α-FLAG
48
35
α-FLAG
48
(Input: 2XFLAG Mts^WT)

Supplementary Figure S1. Mts interacts with conserved domains of Vtd. Related to Figure 1

(a) Vtd^{WT} can be divided into five domains. $Vtd^{1\sim152}$, $Vtd^{273\sim530}$ and $Vtd^{625\sim715}$ are globular domains conserved in Rad21 family proteins. $Vtd^{153\sim272}$ and $Vtd^{531\sim624}$ are not conserved. (b) Alignment of conserved Rad21 protein sequences using ClustalW. Conserved domains ($Vtd^{1\sim152}$, $Vtd^{273\sim530}$ and $Vtd^{625\sim715}$) colored in (a) are indicated by lines. (c) Co-immunoprecipitation assay using S2 cell extract. FLAG-tagged Mts^{WT} forms a protein complex with V5-tagged $Vtd^{1\sim152}$, $Vtd^{273\sim530}$ and $Vtd^{625\sim715}$. The $Vtd^{153-272}$ fragment appears to migrate slower than expected for unknown reason, although the correct sequence of this construct was confirmed. Asterisk indicates a non-specific band which is immunoprecipitated by V5 antibody. This non-specific band overlaps with the Vtd^{WT}-V5/His band. Full-length blots are presented in Supplementary Figure 6.

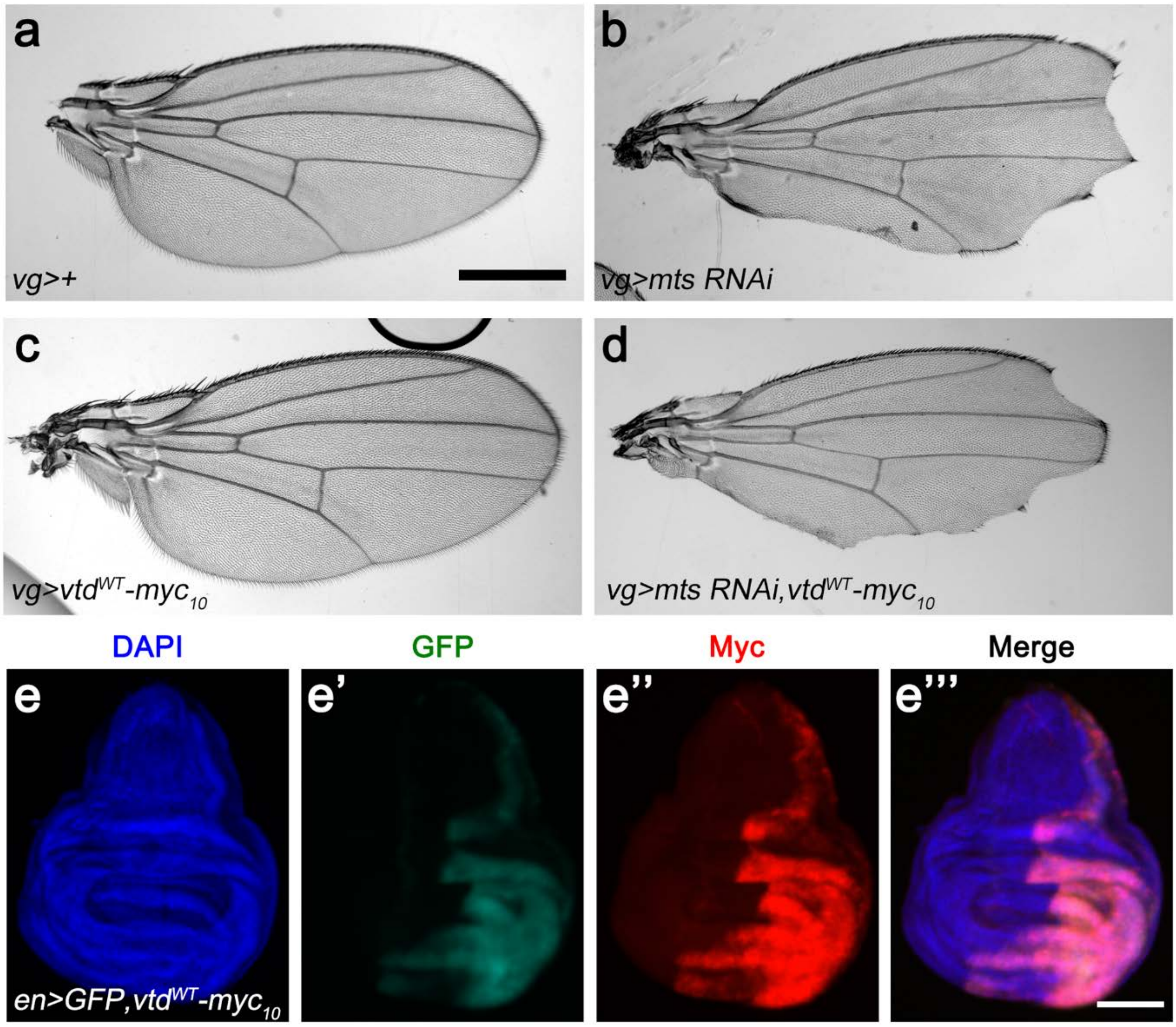

Supplementary Figure S2. Vtd overexpression fails to rescue the *mts* RNAi wing phenotype. Related to Figure 4

(a-d) Female adult wings of indicated genotypes. (a) Control (*vg*>+). (b) *mts* knockdown (*vg*>*mts RNAi*) causes notching phenotype. (c) Vtd^{WT} overexpression (*vg*>vtd^{WT}-myc_{10}) shows normal wing. (d) Vtd^{WT} overexpression fails to rescue the notch phenotype of *mts* RNAi. Scale bar, 100μm. (e-e’’’’) Vtd^{WT}-myc_{10} expression by *en*>*Gal4*. Wing imaginal discs were stained by DAPI (blue) (e), GFP (e’), anti-Myc (e”). (e”’) Merge of e-e”. The posterior compartment of wing disc is marked by *en*>*GFP* (e’). Scale bar, 100μm.

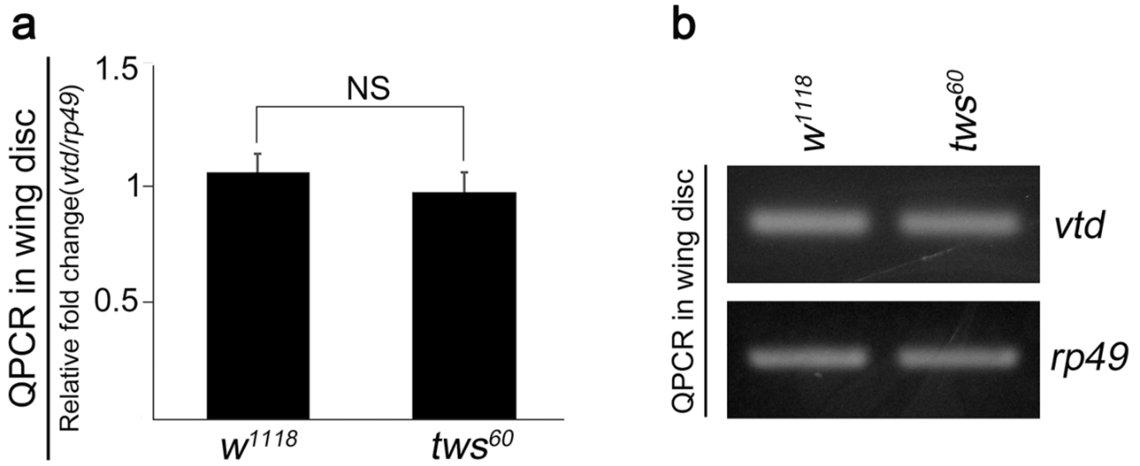

Supplementary Figure S3. Mts is required to maintain the level of Vtd protein but not *vtd* transcription. Related to Figure 5

(a, b) Quantitative PCR analysis (QPCR) in wing imaginal disc tissues. (a) The level of *vtd* transcript is not significantly changed in tws^{60} homozygous mutant. Error bars are s.d (n=3). NS, Not significant (*P*=0.8). (b) A representative gel image of QPCR results. Full-length gels are presented in Supplementary Figure 6.

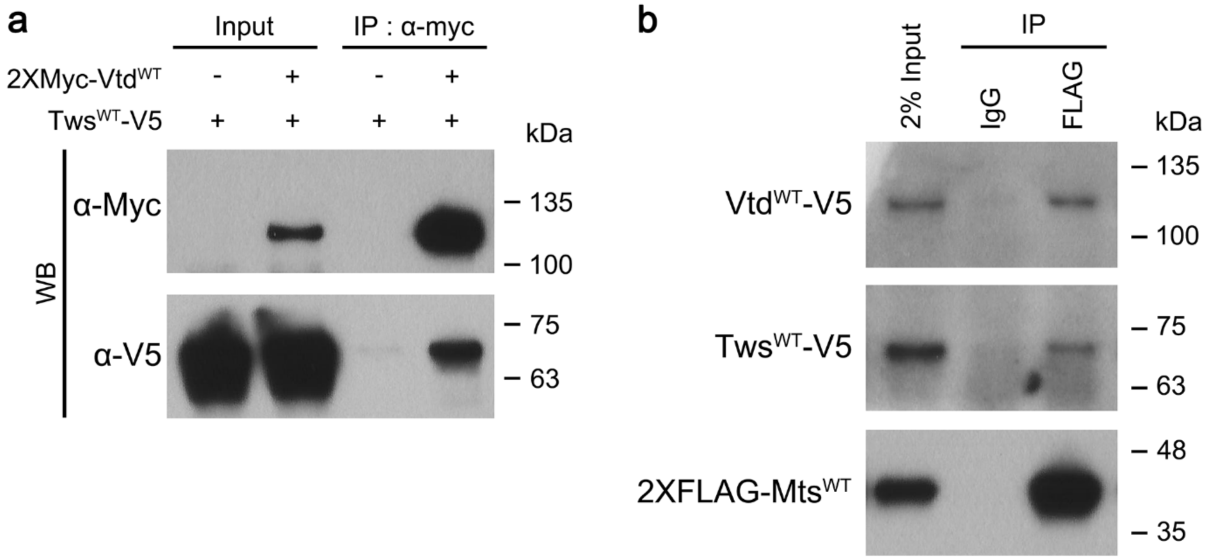

Supplementary Figure S4. Mts co-immunoprecipitates with Tws and Vtd. Related to Figure 5

(a, b) Co-immunoprecipitation assay using S2 cell extracts. Full-length blots are presented in Supplementary Figure 6. (a) 2XMyc-tagged Vtd^{WT} forms a protein complex with V5-tagged Tws^{WT} in S2 cell. (b) 2XFLAG-tagged Mts^{WT} co-immunoprecipitates with V5-tagged Vtd^{WT} and V5-tagged Tws^{WT} in S2 cell.

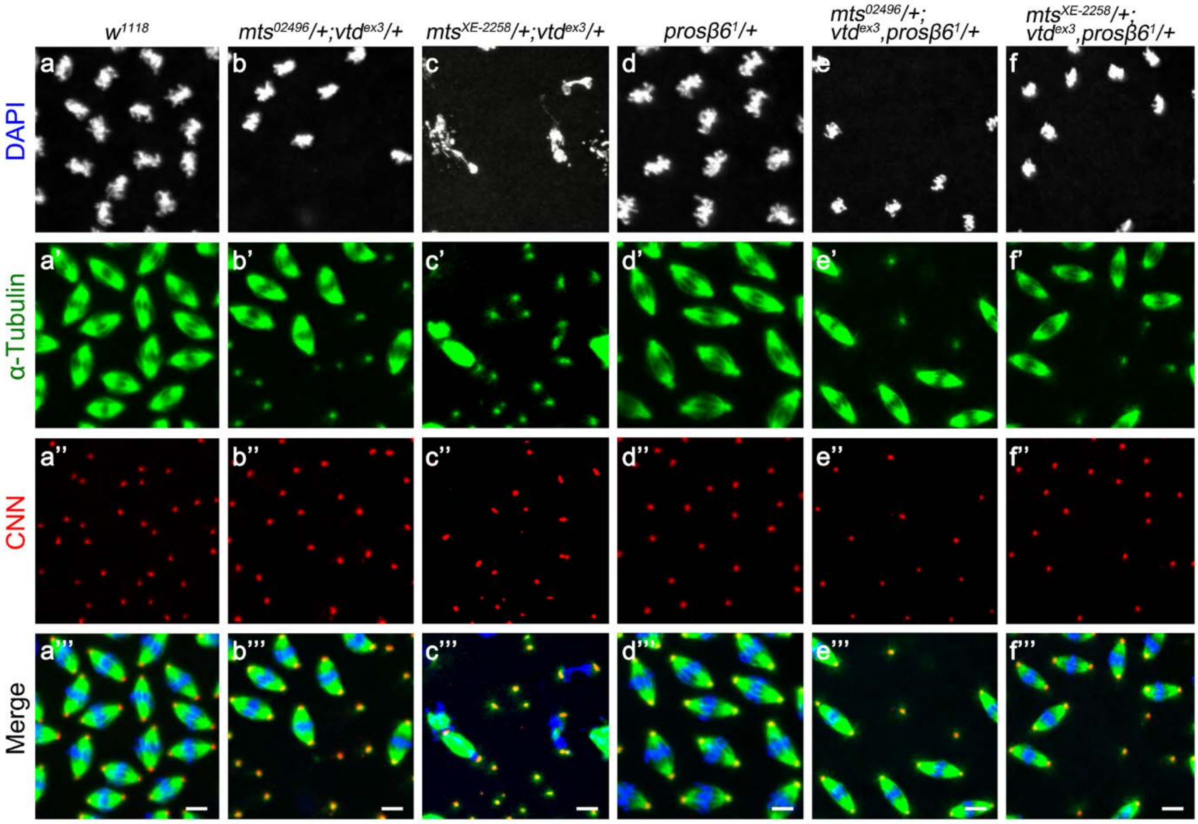

Supplementary Figure S5. Vtd stability is regulated by PP2A through proteasomal degradation pathway. Related to Figure 6.

Figure 6c-h show confocal images with only merged channels. (a-f''') Separate and merged channels of Figure 6c-h are shown as indicated.

Fig.1

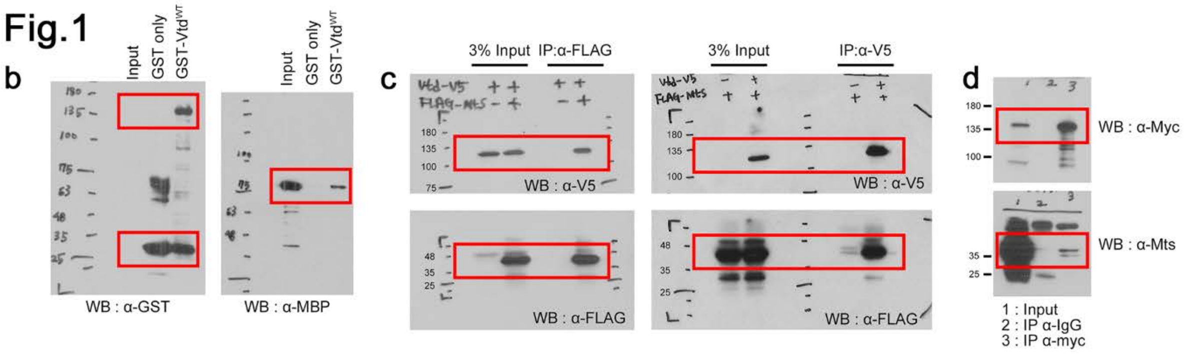

Fig.5

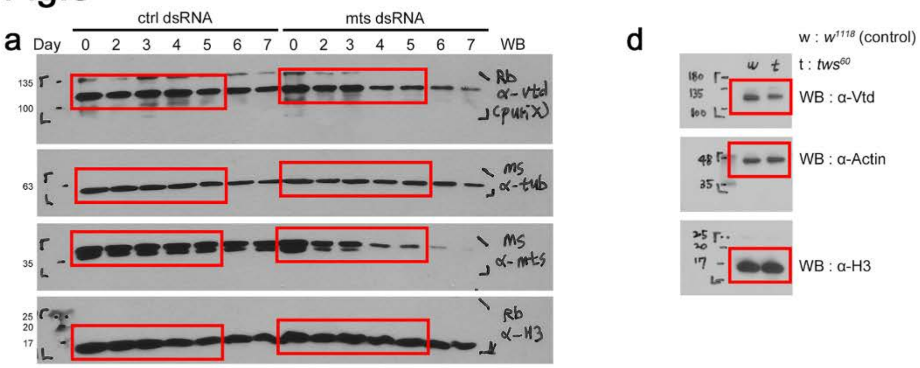

Fig.6

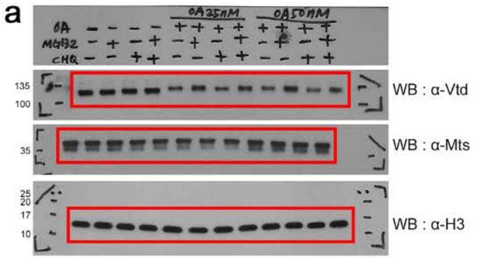

Fig.S1

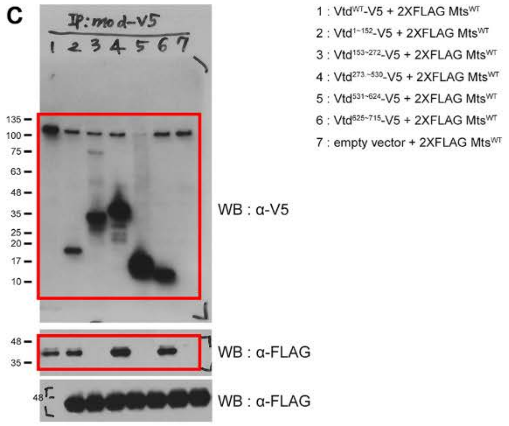

Fig.S4

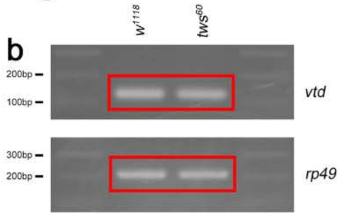

Fig.S5

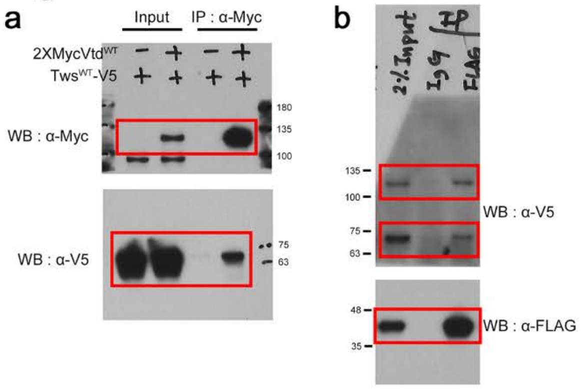

Supplementary Figure S6. Full-length blots and gels presented in the manuscript

Red rectangles are the areas cropped from full-length blots. (Fig. 1b) A blot was made from a single gel. The same blot was immunostained with anti-MBP and restained with anti-GST after stripping. (Fig. 1c) Two blots on the left were made from a single gel. After protein transfer from the gel, the blot was cut into two blots for staining with different antibodies. Two blots on the right were also from a single gel and cut into two for staining with two antibodies as indicated. (Fig. 1d) Two blots shown were from two separate gels and blotted separately. (Fig. 5a, d) Blots in 'a' and 'd' are from two different gels. A blot from each gel was cut as indicated for staining with different antibodies. (Fig. 6a) A blot from a single gel was cut as indicated for staining with different antibodies. (Fig. S1c) A blot from a single gel was cut as indicated for staining with different antibodies. (Fig. S4) RT PCR samples amplified for *vtd* and *rp49* were loaded on the same gel. (Fig. S5a, b) Blots in 'a' and 'b' are from two different gels. A blot from each gel was cut as indicated for staining with different antibodies.
